# Verletzungen der BWS und LWS bei Kindern unter 16 Jahre – Versorgungsrealität im deutschsprachigen Raum: eine Registerstudie

**DOI:** 10.1007/s00113-024-01504-3

**Published:** 2024-12-06

**Authors:** Hauke Rüther, Saleh Alayesh, Christoph-E. Heyde, Dina Wiersbicki, Yasmin Youssef, Julia Bolte, Pia Brecht, Alexander Carl Disch, Jan-Sven Jarvers, Arbeitsgruppe Wirbelsäulentrauma im Kindesalter der Sektion Wirbelsäule der Deutschen Gesellschaft für Orthopädie und Unfallchirurgie (DGOU)

**Affiliations:** 1 Klinik für Unfallchirurgie, Orthopädie und Plastische Chirurgie, Universitätsmedizin Göttingen, Georg-August-Universität, Göttingen, Deutschland; 2 Klinik und Poliklinik für Orthopädie, Unfallchirurgie und Plastische Chirurgie, Universitätsklinikum Leipzig AöR, Leipzig, Deutschland; 3 Klinik für Orthopäde und Unfallchirurgie, Kreiskrankenhaus Torgau „Johann Kentmann“ gGMBH, Christianistraße 1, 04860 Torgau, Deutschland; 4 UniversitätsCentrum für Orthopädie, Unfall- & Plastische Chirurgie, Universitätsklinikum Carl Gustav Carus, Dresden, Deutschland; 5 Klinik für Kinderorthopädie und Kindertraumatologie, Helios Klinikum Emil von Behring GmbH, Berlin, Deutschland

**Keywords:** Multizenterstudie, Dorsale Stabilisierung, Neurologische Defizite, Dekompression, Komplikationsrate, Multicenter study, Dorsal stabilization, Neurological deficits, Decompression, Complication rate

## Abstract

**Hintergrund:**

Wirbelsäulenverletzungen im Kindesalter stellen eine Seltenheit dar. Für den deutschsprachigen Raum gibt es wenige aussagekräftige Daten.

**Fragestellungen/Ziel der Arbeit:**

Auswertung der Registerdaten der Deutschen Wirbelsäulengesellschaft (DWG) im Hinblick auf die Versorgungsrealität von thorakolumbalen Verletzungen im Kindesalter.

**Material und Methoden:**

Diese retrospektive Studie wurde durch die Arbeitsgruppe Wirbelsäulentrauma im Kindesalter der Sektion Wirbelsäule der Deutschen Gesellschaft für Orthopädie und Unfallchirurgie (DGOU) initiiert. Eingeschlossen wurden alle operierten Patienten unter 16 Jahren, die vom 01.01.2017 bis zum 31.06.2023 eingepflegt wurden. Hierbei erfolgte eine anonymisierte Auswertung der demografischen Daten im Hinblick auf Alter, Geschlecht, Verletzungshöhe, neurologische Beeinträchtigung, Klassifikation sowie durchgeführte Therapie mit intra- und postoperativen Komplikationen. Als Informationsgrundlage diente die vorgegebene Dokumentation im Wirbelsäulenregister. Bezüglich der Alterseinteilung wurde die Alterseinteilung in 3 Gruppen verwendet: I: 0 bis 6, II: 7 bis 9, III: 10 bis 16 Jahre.

**Ergebnisse:**

Daten von 83 Kindern mit 150 Verletzungen im Bereich der BWS und LWS mit einem mittleren Alter von 11,4 (± 3,45) Jahren wurden analysiert. Es zeigten sich 78 (52%) thorakale und 72 (48%) lumbale Verletzungen. Am häufigsten traten Typ-A-Verletzungen (*n* = 89; 59,2%) auf. Typ-B-Verletzungen zeigten sich in 32,2% (*n* = 48) und traten v. a. in Gruppe III auf. Gemäß der AO Neurologic Injury Classification zeigten 18 (21%) Patienten inkomplette, 4 (4,8%) Patienten komplette Querschnittslähmungen. Zur operativen Therapie wurden verschiedene Operationsmethoden eingesetzt, am häufigsten war die dorsale Stabilisierung (*n* = 73; 87,9%). Die Mehrheit der Operationen verlief komplikationslos (*n* = 75; 90,4%).

**Diskussion:**

Es wurden laut Registerdaten 83 Kinder mit akzeptabler Komplikationsrate operativ versorgt. Ältere Kinder und Jugendliche (Gruppe III) wiesen eine signifikant höhere Verletzungsschwere im Vergleich zu jüngeren Kindern auf. Wie bei den meisten Registeruntersuchungen lassen auch hier nur begrenzte Schlussfolgerungen über chirurgische Strategien, Indikationen und Techniken ziehen.

## Abstract (en)

**Background:**

Spinal injuries in childhood are rare. There are few meaningful data for German-speaking countries.

**Questions/aim of the study:**

Evaluation of the registry data of the German Spine Society (DWG) with respect to the reality of care for thoracolumbar injuries in childhood.

**Material and methods:**

This retrospective study was initiated by the Spinal Trauma in Childhood Working Group of the Spine Section of the German Society for Orthopaedics and Trauma Surgery (DGOU). All patients under the age of 16 years who underwent surgery between 1 January 2017 and 31 June 2023 were included. An anonymized evaluation of the demographic data was carried out with respect to age, gender, level of injury, neurological impairment, classification and treatment performed with intraoperative and postoperative complications. The specified documentation in the spine register served as the information basis. Regarding age classification 3 groups were used: I: 0–6 years, II: 7–9 years, III: 10–16 years.

**Results:**

Data from 83 children with 150 injuries in the thoracic and lumbar spine areas with a mean age of 11.4 (± 3.45) years were analyzed. A total of 78 (52%) thoracic and 72 (48%) lumbar injuries were found. Type A injuries were most common (*n* = 89; 59.2%). Type B injuries were found in 32.2% (*n* = 48) and occurred mainly in group III. According to the AO neurological injury classification 18 (21%) patients had incomplete and 4 (4.8%) patients had complete paraplegia. Various methods were used for surgical treatment, the most common being dorsal stabilization (*n* = 73; 87.9%). The majority of operations were uncomplicated (*n* = 75; 90.4%).

**Discussion:**

According to the registry data 83 children were treated surgically with an acceptable complication rate. Older children and adolescents (group III) had a significantly higher injury severity compared to younger children. As with most registry studies, only limited conclusions can be drawn about surgical strategies, indications and techniques.

## Einleitung

Mit einem Auftreten von 1–4 % zählen kindliche Wirbelsäulenverletzungen zu den seltenen Traumafolgen [11].

Aufgrund dieser Seltenheit bedarf es einer genauen Kenntnis möglicher Verletzungsformen, der physiologischen Entwicklung anatomischer Strukturen, Varianten und Anomalien, denn nur dann können die Verletzungen erkannt und adäquat behandelt werden [6, 21].

Die Analyse der Inzidenz von Wirbelsäulenverletzungen bei jungen Patienten zeigt signifikante regionale Differenzen auf, sodass eine Übertragung der angloamerikanischen Daten auf den deutschsprachigen Raum nicht angebracht erscheint [10].

Abgesehen von der aktuellen deutschen Multizenterstudie [9] ist es schwer, repräsentative Daten über thorakolumbale Wirbelsäulenverletzungen in Österreich, der Schweiz oder Deutschland zu generieren.

Ziel dieser Arbeit war es daher, die Daten des durch die Deutsche Wirbelsäulengesellschaft (DWG) eigenständig betriebenen Registers im Hinblick auf die Versorgungsrealität von thorakolumbalen Verletzungen im Kindesalter auszuwerten. Eine Aussage über den Verletzungshergang wird allerdings nicht vom Register abgefragt.

Die Teilnahme am Deutschen Wirbelsäulenregister ist obligater Bestandteil der Institutszertifizierung durch die DWG, und die Rechte und Pflichten der Teilnehmer am Deutschen Wirbelsäulenregister sind durch Teilnahmebedingungen verbindlich geregelt.

## Material und Methoden

Diese retrospektive Studie wurde durch die Arbeitsgruppe Wirbelsäulentrauma im Kindesalter der Sektion Wirbelsäule der Deutschen Gesellschaft für Orthopädie und Unfallchirurgie (DGOU) initiiert. Nach entsprechender Antragsstellung erfolgte die Genehmigung des Vorstandes der DWG zur Auswertung der Registerdaten.

Die statistische Auswertung der Daten erfolgte durch SwissRDL (www.swissrdl.unibe.ch) der Universität Bern, durch welche das Deutsche Wirbelsäulenregister und die Datenauswertung im Rahmen des Registers technisch betreut wurden.

Eingeschlossen wurden alle operierten Patienten unter 16 Jahren, die vom 01.01.2017 bis zum 31.06.2023 in das Register eingepflegt wurden.

Hierbei erfolgte eine anonymisierte Auswertung der demografischen Daten im Hinblick auf Alter, Geschlecht, Verletzungshöhe, neurologische Beeinträchtigung, Klassifikation sowie durchgeführte Therapie mit intra- und postoperativen Komplikationen. Als Informationsgrundlage diente die vorgegebene Dokumentation im Wirbelsäulenregister.

Bezüglich der Alterseinteilung wurde die aus Jarvers et al. [10] bekannte Alterseinteilung in 3 Gruppen verwendet:

- I: 0 bis 6 Jahre,
- II: 7 bis 9 Jahre
- III: 10 bis 16 Jahre.

## Ergebnisse

Insgesamt konnten Daten von 83 Kindern mit 150 Verletzungen im Bereich der BWS und LWS aus dem genannten Zeitraum analysiert werden. Das mittlere Alter zum Zeitpunkt der Verletzung betrug 11,4 (± 3,45) Jahre. Die Altersgruppe III der Jugendlichen und Adoleszenten stellte mit 71 Patienten (85,5 %) die stärkste Gruppe dar.

### Geschlechterverteilung

**Abb. 1:**
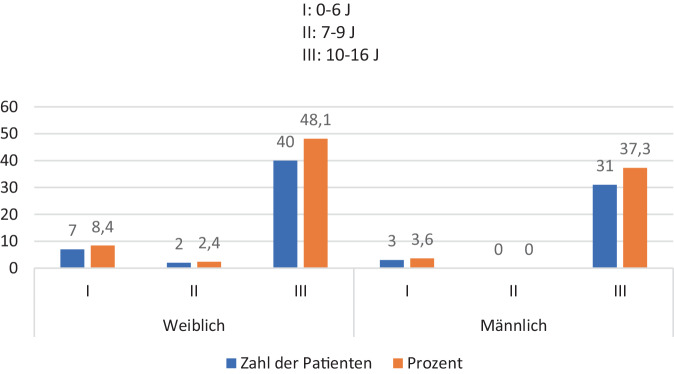
Geschlechterverteilung auf die Altersgruppen

Es verletzten sich weniger Jungen (*n* = 34; 40,9 %) als Mädchen (*n* = 49; 59,1 %). Das Verhältnis betrug 1:1,44 (Abb. 1).

### Lokalisation der Verletzung

**Abb. 2:**
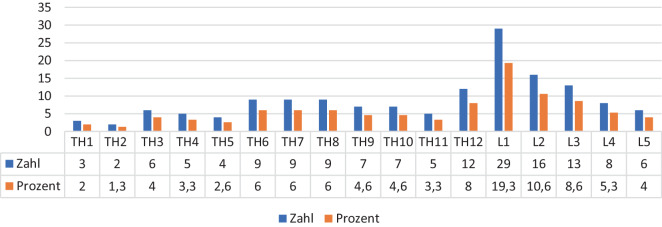
Anzahl und Lokalisation der Frakturen (alle Altersgruppen)

Bei den 83 Patienten wurden insgesamt 150 Verletzungen der Wirbelsäule diagnostiziert. Es stellten sich 78 (52 %) thorakale und 72 (48 %) lumbale Wirbelsäulenverletzungen dar. Die Detailanalyse der Verletzungshöhe ergab eine Häufung am thorakolumbalen Übergang. Daneben waren an der BWS der 6. bis 8. Wirbelkörper betroffen. Unter Berücksichtigung der Altersgruppen zeigte sich für die BWS, dass Patienten der Gruppe I signifikant häufiger Verletzungen an der oberen BWS aufwiesen als Jugendliche und Adoleszente (Gruppe III). Dahingegen waren Verletzungen im thorakolumbalen Übergang im Vergleich zur oberen BWS und zur unteren LWS mit einem höheren Patientenalter (Gruppe III) assoziiert. In jedem Wirbelsäulenabschnitt nahm die Zahl der schweren Verletzungen mit dem Alter des Kindes zu. Von den 83 Patienten hatten 21 (25,3 %) eine isolierte Fraktur, während die übrigen 62 (74,5 %) Patienten 2 oder mehrere Frakturen aufwiesen (Abb. 2).

**Abb. 3:**
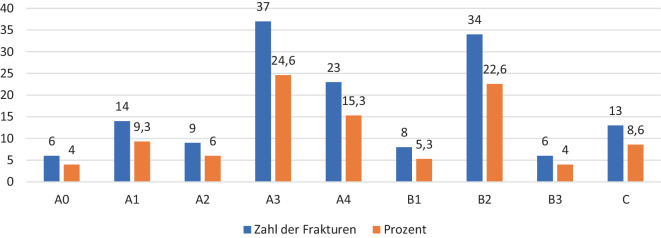
Klassifikation der Verletzungen

Der Anteil von Typ-C-Frakturen nach der AO-Spine-Klassifikation an der BWS und LWS (*n* = 13; 8,6 %) war im Vergleich zu Typ-A- und Typ-B-Frakturen gering. Am häufigsten zeigten sich Typ-A-Verletzungen (*n* = 89; 59,2 %), welche im BWS- und im LWS-Bereich gleichmäßig auftraten. Typ-B-Verletzungen zeigten sich in 32,2 % (*n* = 48) und traten v. a. bei älteren Kinder und Adoleszenten auf (Gruppe III) (Abb. 3).

**Abb. 4:**
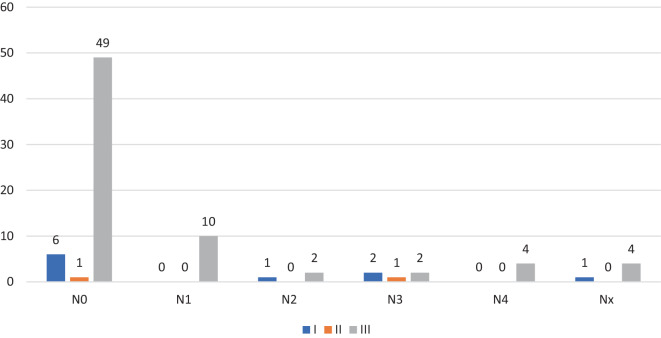
Neurologische Defizite in Bezug auf Altersgruppen

Neurologische Ausfälle wurden nur selten beobachtet. Gemäß der AO Neurologic Injury Classification [3] ließen sich 56 Patienten der Grad N0 (67,4 %), 10 Patienten der Grad N1 (12 %), 3 Patienten der Grad N2 (3,6 %) und 5 Patienten der Grad N3 (6 %) zuordnen. Komplette Querschnittslähmungen N4 wurden bei 4 Patienten dokumentiert (4,8 %) (Abb. 4 und 5). Je komplexer die Wirbelsäulenverletzung, desto höher der Grad des neurologischen Defizits.

**Abb. 5:**
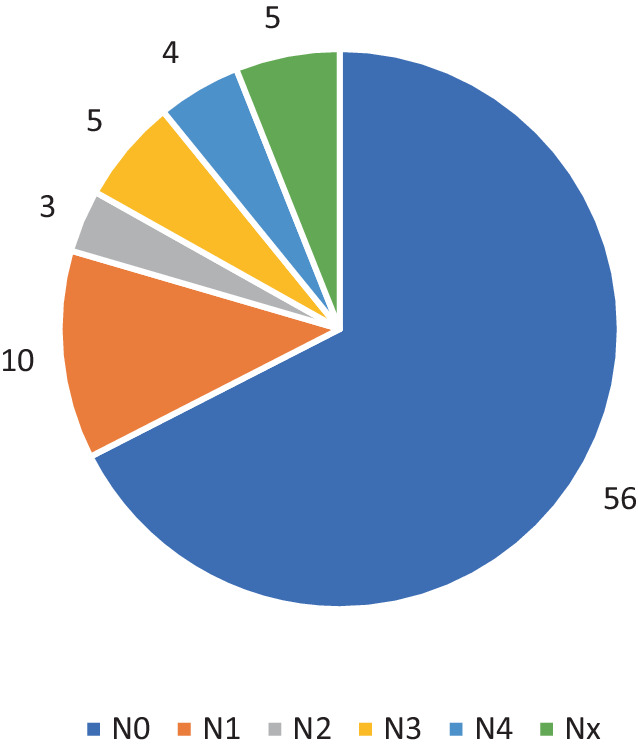
Neurologische Defizite nach AO Neurologic Injury – Classification [3]

### Hospitalisierung

Die mittlere Krankenhausverweildauer betrug 18,3 (± 0,85) Tage, wobei der kürzeste Aufenthalt 7 Tage und der längste Aufenthalt 61 Tage umfasste. Erwartungsgemäß führte die operative Therapie, die Verletzungsschwere oder das Vorliegen von neurologischen Defiziten zu einer signifikant verlängerten Hospitalisierungsdauer. Der stationäre Aufenthalt in der Gruppe I betrug durchschnittlich 19,1 (± 3,4) Tage. Deutlich kürzer verweilten die Kinder der Gruppe II im Krankenhaus mit 17,4 (± 3,61) Tagen. Die durchschnittliche Hospitalisierung der Gruppe III dauerte 18,4 (± 3,1), Tage. Bei 5 Patienten dauerte der Aufenthalt auf der Intensivstation mehr als 3 Tage.

Bei 4 Patienten traten die Wirbelsäulenverletzungen als Begleitverletzungen im Rahmen eines Polytraumas auf, was zu einem verlängerten stationären Aufenthalt führte.

**Abb. 6:**
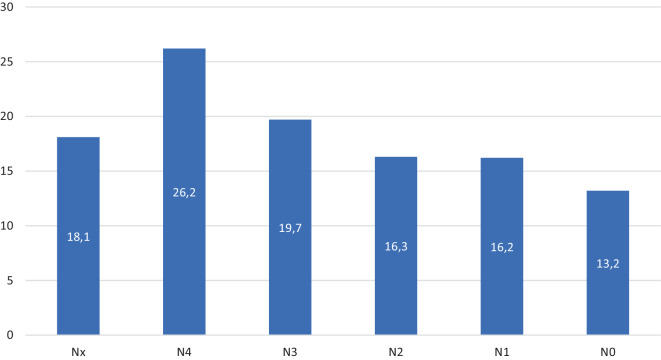
Durchschnittliche Verweildauer (in Tagen) in Bezug auf neurologische Ausfälle

Je schwerwiegender die Wirbelsäulenverletzung war, desto ausgeprägter war das neurologische Defizit, was zu einer längeren stationären Behandlungsdauer der Patienten führte (Abb. 6).

### Therapie

**Abb. 7:**
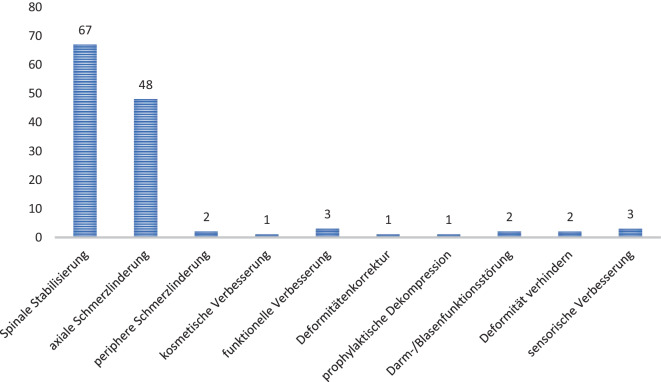
Angegebene Operationsziele

In der Gesamtkohorte wurden 83 Patienten mit 150 Frakturen operativ behandelt. Angegebene Hauptziele der Operation waren die Wirbelsäulenstabilisierung und Schmerzlinderung. Als sekundäres Ziel wurde die Verbesserung der Motorik und Sensibilitäten genannt (Abb. 7).

Bei 18,5 % (*n* = 18) der Patienten dauerte die Operation weniger als 1 h. Bei 37 % (*n* = 38) betrug die Operationszeit bis zu 2 h, während sie bei 26 % (*n* = 22) zwischen 2 und 3 h lag. Bei weiteren 18,5 % dauerte die Operation mehr als 3 h.

**Abb. 8:**
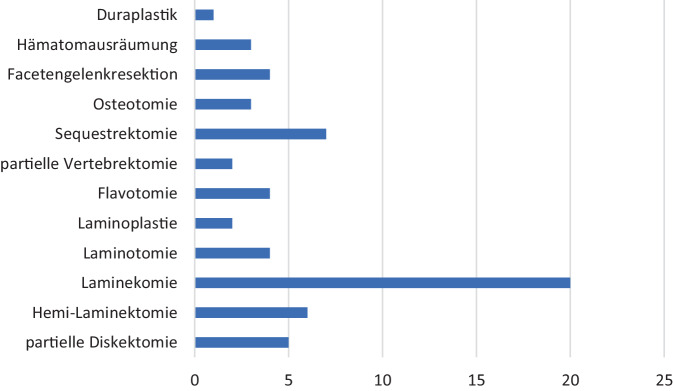
Häufigkeit der Dekompressionsverfahren

**Abb. 9:**
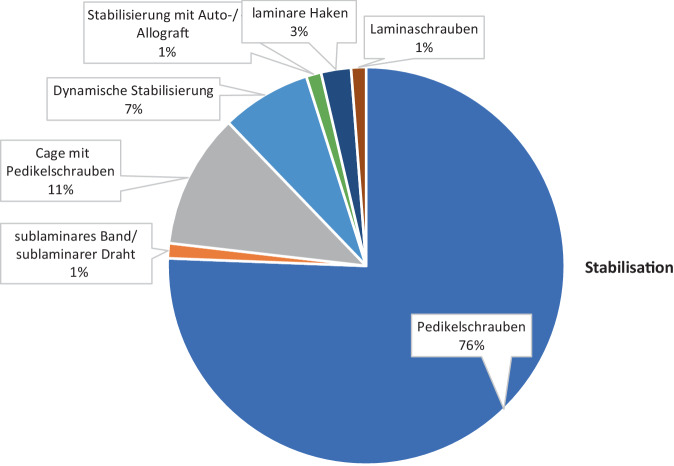
Stabilisierungsverfahren

Zur operativen Therapie wurden verschiedene Operationsmethoden eingesetzt. In 33 Fällen (39 %) wurde u. a. eine Dekompression durchgeführt. Hier ergab sich eine Signifikanz bezüglich der Altersverteilung. Gruppe III wurden 26 Patienten (*n* = 26; 78,7 %) wurden zugeordnet. Fünf Patienten gehörten zur Gruppe I (*n* = 5; 15,1 %) und 2 Patienten zur Gruppe II (*n* = 2; 6,2 %). Die Laminektomie war der am häufigsten durchgeführte Eingriff, bezogen auf die Dekompression (*n* = 20; 32,7 %). An zweiter Stelle stand die Sequestrektomie mit (*n* = 7; 11,4 %). Die Hemilaminektomie war mit 6 Fällen (*n* = 6; 9,8 %) die dritthäufigste Dekompression. Die Diskektomie erfolgte bei 5 Patienten (8,1 %). Die Laminotomie, Facettengelenkresektion und Flavotomie erfolgte jeweils bei 4 Patienten (6,5 %). Des Weiteren wurde bei 2 Kindern eine Vertebrektomie (*n* = 2; 3,2 %) durchgeführt. Einmal wurde eine Duraplastik (*n* = 1, 1,6 %) durchgeführt (Abb. 9). Die meisten Dekompressionseingriffe erfolgten im thorakolumbalen Übergang, insbesondere an den Wirbelkörpern BWK 12, LWK 1 und LWK 2 (*n* = 26; 37,1 %) (Abb. 8).

In 19 Fällen wurde eine Fusion indiziert, hier ergab sich keine Signifikanz bezüglich der Altersverteilung und der Lokalisation der Frakturen. In 12 Fällen erfolgte eine posteriore (63,1 %), in 7 Fällen (36,8 %) posterolaterale Fusion (Abb. 9).

Der am häufigsten durchgeführte Eingriff, sowohl mit als auch ohne Dekompression, war die dorsale Stabilisierung (*n* = 73; 87,9 %). Hiervon wurden bei 61 Verletzungen (83,5 %) minimalinvasive Stabilisierungen mit Pedikelschrauben durchgeführt, während bei 12 Patienten (16,5 %) eine konventionell offene Operation durchgeführt wurde. In 9 Fällen wurden ein Cage und Pedikelschrauben implantiert (11 %). Bei 6 Patienten wurden dynamische Stabilisierungen durchgeführt (7 %). Laminahaken wurden in 2 Fällen (*n* = 2; 3 %), jeweils einmalig Laminaschrauben und ein sublaminares Band/sublaminarer Draht (1,3 %) zur Fixation genutzt.

Der thorakolumbale Übergang wurde am häufigsten stabilisiert. Dabei gehörte der größte Teil der Patienten der Gruppe III (*n* = 65; 89 %) an. Fünf Patienten wurden der Gruppe I (6,8 %) und 3 Patienten der Gruppe II (4,2 %) zugeordnet.

### Verlauf und Komplikation

**Abb. 10:**
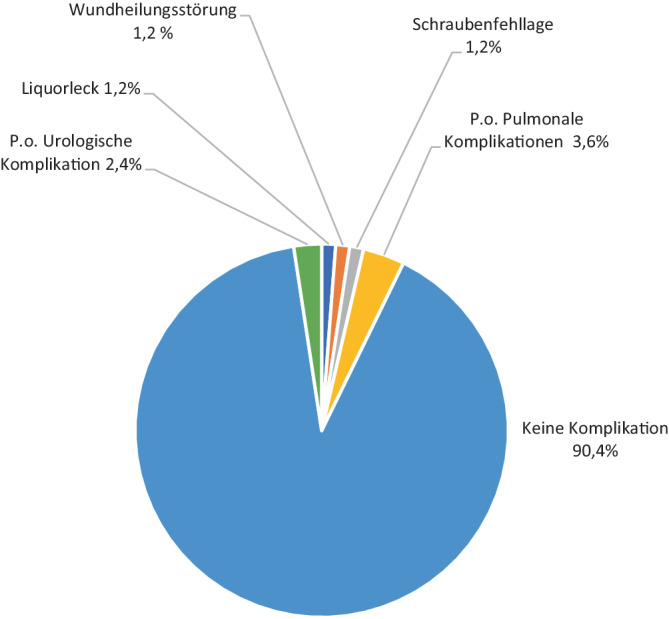
Komplikation

Die Mehrheit der Operationen verlief komplikationslos (*n* = 75; 90,4 %). Bei 3 Patienten (3,6 %) traten postoperative pulmonale Komplikationen auf (2 aus der Gruppe III und einer aus der Gruppe I). Bei jeweils einem Patienten wurden eine Fehlplatzierung der Schrauben, eine Wundheilungsstörung sowie ein Liquorleck festgestellt (1,2 %), die eine Revision erforderlich machten (alle aus Gruppe III). Darüber hinaus entwickelten 2 Patienten postoperative urologische Komplikationen (2,4 %), hier stammen die Patienten auch aus Gruppe III (Abb. 10).

In Zusammenschau der Komplikationen und neurologischen Ausfälle zeigen die Patienten mit urologischen Komplikationen Ausfälle gemäß N3 und N4. Der Patienten mit Liquorleck hatte neurologische Ausfälle gemäß N2.

## Diskussion

Aufgrund des insgesamt seltenen Auftretens von Wirbelsäulenverletzungen in diesem Altersgefüge fehlt es den erstversorgenden Behandlern oft an nötigen Kenntnissen und Vorerfahrungen, um die betroffenen Kinder zeitnah den notwendigen diagnostischen Schritten und der umgehenden Behandlung zuzuführen. Klinisch epidemiologische Daten können helfen, die Versorgung kindlicher Wirbelsäulenverletzungen zu verbessern, allerdings beschränkt sich die Literatur zumeist auf Daten aus monozentrischen Fallserien mit niedrigem Evidenzlevel. Ziel der vorliegenden Analyse war daher, im Rahmen dieser retrospektiven Auswertung des Wirbelsäulenregisters der DWG einen weiteren Einblick in die Versorgungsrealität von Verletzungen in der kindlichen thorakalen und lumbalen Wirbelsäule zu geben.

In der entsprechenden Auswertung der Registerdaten konnten im Zeitraum von Januar 2017 bis Juni 2023 83 Kinder mit 150 Verletzungen an der thorakolumbalen Wirbelsäule eingeschlossen werden. Demgegenüber stehen in der aktuellen Multizenterstudie der AG kindliches Wirbelsäulentrauma der DGOU 153 Kinder mit 345 Verletzungen an der thorakolumbalen Wirbelsäule [9]. Im Zeitraum Januar 2010 bis Dezember 2016 wurden 58 operiert. Auch wenn der Zeitraum nicht exakt identisch ist, fällt doch die insgesamt geringe Anzahl der dokumentiert operierten Patienten in einem Zeitraum von über 12 Jahren auf. Im Vergleich zur Multizenterstudie, an der 6 Wirbelsäulenzentren teilgenommen hatten, war die Anzahl der am Register teilnehmenden Kliniken größer. Dies zeigt einmal mehr die Seltenheit dieser Verletzungsentität, unterstreicht aber auch die Problematik der Registerdatenerhebung, zumal mit Sicherheit eine weit höhere Anzahl von operativen Versorgungen von Verletzungen dieser Art im deutschsprachigen Raum stattgefunden hat.

Während der thorakolumbale Übergang bei letzterer Studie die häufigste Lokalisation der Verletzung bei allen Patienten war, zeigten ältere Kinder und Jugendliche der Gruppe III häufig Verletzungen im Bereich der mittleren Brustwirbelsäule [10].

Ein ähnliches Ergebnis zeigt die durchgeführte Registerstudie. Die meisten Verletzungen im thorakolumbalen Übergang waren hier bei LWK 1 lokalisiert. Unter Berücksichtigung der Altersgruppen zeigte sich bei den Registerdaten jedoch im Gegensatz dazu, dass Kinder der Gruppe I signifikant häufiger Verletzungen an der oberen BWS aufwiesen als Jugendliche und Adoleszente (Gruppe III). Dahingegen waren Verletzungen im thorakolumbalen Übergang im Vergleich zur oberen BWS und zur unteren LWS mit einem höheren Patientenalter assoziiert. In jedem WS-Abschnitt nahm die Zahl der schweren Verletzungen mit dem Alter des Kindes zu.

Das durchschnittliche Alter der Multizenterstudie betrug 12,9 (± 3,1) Jahre und zeigte sich damals vergleichbar mit den Ergebnissen von Kraus et al. [12]. In den aktuellen Registerdaten zeigte sich ein niedrigeres Durchschnittsalter von 11,4 (± 3,2) Jahren.

Das Geschlechterverhältnis betrug 1,44:1 (weiblich/männlich). Im Gegensatz dazu ergab die Multizenterstudie eine homogenere Geschlechterverteilung von 1:1,3. Aussagen in anderen Studien, dass fast ausschließlich Jungen eine Wirbelsäulenverletzung erleiden, konnten für die beiden Kohorten nicht verifiziert werden [9, 14].

Das Risiko nichtzusammenhängender Wirbelsäulenmehrfachverletzungen ist bei Erwachsenen gut belegt und schwankt zwischen 4 und 11 % [4].

Diese Ergebnisse konnten in der deutschen Multizenterstudie nicht auf Kinder übertragen werden [9]; es zeigten sich lediglich bei 1,2 % der Patienten zusätzliche Verletzungen in einem anderen Wirbelsäulensegment. Laut Literatur sind bei Kindern benachbarte Wirbel häufiger betroffen [15]. Dies bestätigt sich auch bei den aktuellen Registerdaten: Von den 83 Patienten wiesen 21 (25,3 %) eine isolierte segmentbezogene Fraktur auf, während die übrigen 62 (74,5 %) Patienten 2 oder mehrere Frakturen aufwiesen. Diese seriellen Frakturen sind v. a. bei kleineren Kindern typisch und äußern sich v. a. in der TIRM- oder STIR-Sequenz im MRT als okkulte Frakturen bzw. Ödeme [22]. Da sie in der Regel keiner Operation bedürfen, wurden sie auch nicht vom Register erschlossen, womit die Zahl nicht die komplette Realität dieser Verletzungen darstellt.

Der Anteil von Typ-C-Frakturen nach der AO-Spine-Klassifikation an der BWS und LWS (*n* = 13; 8,6 %) war im Vergleich zu A‑ und B‑Frakturen erwartungsgemäß gering. Am häufigsten zeigten sich über alle Altersgruppen Typ-A-Verletzungen (*n* = 89; 59,2 %), die an der BWS und LWS gleich verteilt auftraten. Typ-B-Verletzungen zeigten sich in 32,2 % (*n* = 48) und traten v. a. bei älteren Kinder und Adoleszenten der Gruppe III auf.

Vergleichbare Ergebnisse konnten in der deutschen Multizenterstudie [9] gezeigt werden. Der häufigste Frakturtyp in dieser Kohorte war mit 87 % der Typ A nach der AO-Spine-Klassifikation. Obwohl in der Literatur ein hoher Anteil von B‑Verletzungen (insbesondere Typ B2) beschrieben wird [20], war der Anteil in dieser Kohorte mit 5,6 % eher gering.

Der Anteil der Kinder mit Wirbelsäulentrauma und gleichzeitigen neurologischen Verschlechterungen liegt in der Literatur zwischen 2,5 und 35 % [13]. Insgesamt sind schwere neurologische Defizite bei Kindern selten und machen nur 2–8 % der Gesamtzahl der Zahl der Querschnittsgelähmten aus [19]. Auch in der Studie von Herren et al. [9] waren die neurologischen Defizite mit 9 % selten. Dies deckt sich nicht ganz mit den Registerdaten. Gemäß der AO Neurologic Injury Classification ließ sich bei 56 Patienten der Grad N0 (67,4 %), bei 10 Patienten Grad N1 (12 %), bei 3 Patienten Grad N2 (3,6 %) und bei 5 Patienten Grad N3 (6 %) zuordnen. Komplette Querschnittslähmungen N4 wurden bei 4 Patienten dokumentiert (4,8 %). Je schwerwiegender die Wirbelsäulenverletzung war, desto höher war auch der Grad des neurologischen Defizits. Ein höheres Alter der Patienten war auch mit einem schweren neurologischen Defizit assoziiert. Erwartungsgemäß verblieben Patienten mit neurologischem Defizit aufgrund des erhöhten Pflegebedarfs und der zu organisierenden Anschlussheilbehandlung bzw. Verlegung in entsprechende Querschnittszentren auch folgerichtig länger in einer stationären Behandlung. Das grundlegende Ziel der Behandlung von Verletzungen der BWS und LWS bei Kindern ist vergleichbar mit dem bei Erwachsenen. Dabei maßgeblich sind die Wiederherstellung von Stabilität, Alignment und der Schutz bzw. die Wiederherstellung der neurologischen Funktion [18]. In den Publikationen dieser Arbeitsgruppe wurden als Hauptindikationen für die chirurgische Behandlung Fehlstellungen in der frontalen und sagittalen Ebene, vollständige korporale Berstungsfrakturen (Typ-A4-Frakturen), B‑/C-Frakturen und das Vorhandensein von neurologischen Beeinträchtigungen postuliert [17].

Neben der bekannten offenen Instrumentation mit oder ohne Dekompression hat die minimalinvasive perkutane Pedikelschraubenplatzierung bei Erwachsenen vielversprechende Ergebnisse zeigen können [7, 8, 16]. Diese Technik hat die Vorteile von geringerem Blutverlust, reduzierter Operationszeit, verbunden mit einem verkürzten Krankenhausaufenthalt und einer vergleichbaren Anzahl von Komplikationen [8, 19]. Trotz zahlreicher Berichte über die minimalinvasive Stabilisierung in erwachsenen Kollektiven sind nur wenige Ergebnisse bei Kindern publiziert [2].

Auch wenn es sich bei Typ-C-Verletzungen um sehr instabile Verletzungen handelt, wurden 4 Patienten der deutschen Multizenterstudie minimalinvasiv versorgt. Cui et al. [5] konnten zeigen, dass eine temporäre fusionslose Instrumentierung eine erfolgreiche Behandlung von Verletzungen der thorakolumbalen Wirbelsäule auch bei den Verletzungen der Typen B und C bei pädiatrischen Traumapatienten bieten kann [5]. In den hier präsentierten Registerdaten setzt sich dieser Trend fort: 61 Verletzungen (*n* = 61; 83,5 %) wurde minimalinvasiv stabilisiert, während 12 Patienten (16,5 %) eine konventionell offene Operation erhielten. In 9 Fällen wurde eine dorsoventrale operative Strategie durchgeführt mit Pedikelschrauben und Cage-Implantation (*n* = 9 ; 11 %).

Generell sind die Prinzipien der dorsalen Instrumentierung in der chirurgischen Behandlung von Erwachsenen auf ein jüngeres Patientenkollektiv übertragbar. Auch in den jüngeren Altersgruppen I (0 bis 6 Jahre) und II (7 bis 9 Jahre) ist eine chirurgische Stabilisierung im Falle einer bestehenden Indikation möglich. Allerdings machen die kleineren Proportionen und das Verankerungsverhalten die Eingriffe anspruchsvoll, und die Ergebnisse können nicht auf allgemeine Empfehlungen umgeschrieben werden. Somit bleibt es meist eine fallindividuelle Entscheidung. In den aktuellen Daten finden sich daher die Verwendung von laminaren Haken in 2 Fällen (*n* = 2; 3 %) und jeweils einmalig Laminaschrauben und ein sublaminares Band/sublaminarer Draht (1,3 %) in diesen jüngeren Altersgruppen.

Auffällig ist die hohe Anzahl von Dekompressionen im Patientenkollektiv: Bei 33 Fällen wurde u. a. eine Dekompression durchgeführt. Hier ergab sich eine Signifikanz bezüglich der Altersverteilung: 26 Patienten (*n* = 26; 78,7 %) wurden der Gruppe III zugeordnet; fünf Patienten gehörten zur Gruppe I (*n* = 5; 15,1 %) und 2 Patienten zur Gruppe II (*n* = 2; 6,2 %). Bei den meisten Patienten war die Laminektomie die am häufigsten durchgeführte Dekompression (*n* = 20; 32,7 %). Von diesen zeigten 11 Patienten neurologische Ausfälle: vier N1, einer N2, drei N3 und drei N4. Zwei Patienten stammen aus der Gruppe II und 9 aus der Gruppe III. Alle Patienten, die laminektomiert wurden, wurden auch dorsal stabilisiert.

Bei den meisten Patienten traten während des Krankenhausaufenthalts keine Komplikationen auf (*n* = 75; 90,4 %). Die direkt mit der Operation verbundenen Komplikationen umfassten Wundheilungsstörungen, Schraubenfehllage und Duraverletzungen, jeweils mit einer Häufigkeit eines Falles (1,2 %). Indirekte Komplikationen waren pulmonale Komplikationen, insbesondere Pneumonien mit 3 Fällen (3,6 %) sowie urologische Komplikationen im Sinne von Harnwegsinfektion, die in 2 Fällen (2,4 %) auftraten.

Leicht höhere Werte zeigte die Multizenterstudie von Herren et al. [9]: während 96,7 % (*n* = 153) der Patienten keine Komplikationen zeigten, traten in 5 Fällen (8,6 %) operationsbezogene Komplikationen auf: Zwei Patienten (3,4 %) zeigten ein frühes Implantatversagen, in 2 Fällen (3,4 %) zeigte sich eine oberflächliche Wundinfektion. Bei einem Fall (1,7 %) zeigte sich eine segmentale Hyperkyphose nach einem Verlauf von 3 Monaten, wobei eine dorsoventrale Revision notwendig war.

Geht man bei den Registerdaten zusammengefasst jedoch von einer Anzahl von 83 operativ behandelten Kindern aus, bleibt die Häufigkeit der Komplikationen relativ niedrig (*n* = 3/83, 3,6 %). Obwohl internationale Standards für Diagnostik und Therapie fehlen, scheint das Risiko von chirurgischen Eingriffen offenbar vergleichsweise niedrig zu sein.

Die durchgeführte Studie weist mehrere Limitationen auf. Das retrospektive Studiendesign schränkt die Analyse natürlich ein, da auch eine Auswertung der Behandlungsergebnisse auf Grundlage der vorliegenden Daten nicht möglich ist. Aufgrund der Seltenheit der Verletzung war es nicht realisierbar, eine ausreichend große Fallzahl zu generieren, um statistisch valide Subgruppenanalysen der einzelnen Verletzungsraten und -arten in Abhängigkeit vom Alter der Patienten durchzuführen. Daher lassen sich wie bei den meisten Registeruntersuchungen auch hier nur begrenzte Schlussfolgerung über chirurgische Strategien, Indikationen und Techniken ziehen. Die konservativ behandelten Patienten wurden zudem im Register nicht eingegeben.

Für die klinische Versorgung fehlen sowohl nationale als auch internationale Leitlinien. Es mangelt an evidenzbasierten standardisierten Diagnostik- und Behandlungsalgorithmen, die insbesondere bei der Behandlung seltener Verletzungen zwingend erforderlich sind. Das neu aufgelegte Wirbelsäulenregister wird zeigen, ob in Zukunft hier mehr Aussagen zu oben genannten Kritikpunkten getätigt werden können.

## Fazit für die Praxis

- Thorakolumbale Wirbelsäulenverletzungen im Kindesalter betreffen Mädchen und Jungen zu nahezu gleichen Teilen. Durch die Unfallmechanismen typisch betroffene Abschnitte befinden sich im thorakalen Übergang. Ältere Kinder und Jugendliche (Gruppe III) wiesen eine signifikant höhere Verletzungsschwere im Vergleich zu jüngeren Kindern auf. Die begleitenden neurologischen Defizite zeigen sich erwartungsgemäß bei höhergradigen Wirbelsäulentraumata, was die allgemeine Schwere der Verletzungen widerspiegelt.
- Chirurgische Konzepte für Erwachsene können häufig auch bei älteren Kindern angewendet werden, während jüngere Kinder oft ein individuelles Therapiekonzept erfordern. Es wurden laut Registerdaten 83 Kinder mit akzeptabler Komplikationsrate operativ versorgt.

## Data Availability

Die in dieser Studie erhobenen Datensätze können auf begründete Anfrage beim Korrespondenzautor angefordert werden.
